# Tumor and tumorlike conditions of the pleura and juxtapleural region: review of imaging findings

**DOI:** 10.1186/s13244-021-01038-x

**Published:** 2021-07-08

**Authors:** Julie Desimpel, Filip M. Vanhoenacker, Laurens Carp, Annemiek Snoeckx

**Affiliations:** 1 Department of Radiology, Antwerp University Hospital and University of Antwerp, Drie Eikenstraat 655, 2650 Edegem, Belgium; 2 Department of Radiology, AZ Sint-Maarten, Liersesteenweg 435, 2800 Mechelen, Belgium; 3 Department of Nuclear Medicine, Antwerp University Hospital and University of Antwerp, Drie Eikenstraat 655, 2650 Edegem, Belgium

**Keywords:** (Juxta)pleural lesions, CT, MRI

## Abstract

Pleural lesions form a diagnostic challenge for the radiologist. Whereas lesions can be initially detected on chest radiographs, CT and MRI imaging are the imaging modalities of choice for further characterization. In a number of cases, imaging findings can be relatively specific. In general unfortunately, imaging findings are rather aspecific. Evolution and extrathoracic imaging findings are important clues toward the diagnosis.

## Key points

- CT and MRI are the modalities of choice for imaging (juxta)pleural tumors.
- Imaging findings may be aspecific and diagnostically challenging.
- Patient characteristics and evolution in time are important clues toward the diagnosis.

## Introduction

The thoracic space is divided into right and left pleural cavity with a serous membrane, the pleura, contouring both cavities. The pleura is composed of two thin layers of mesothelium: the visceral and parietal pleura. The visceral pleura covers the surface of the lungs and fissures, whereas the parietal pleura covers the thoracic wall and diaphragm. In most cases, the lung does not completely fill the pleural cavity, allowing the parietal pleurae to converge, forming the pleural recess. The visceral pleura is vascularized by arterial branches of the bronchial and pulmonary arterial system, whereas the parietal pleura by intercostal vessels (systemic supply). Due to intercostal nerve innervation, the parietal pleura is sensitive to pain. Both pleurae form a two-layered pleural space containing approximately 5 ml of physiological pleural fluid. Histologically, the pleura is defined as a serous membrane composed of mesothelial cells and loose connective tissue [1, 2].

There is a broad variation in pleural pathologies, with both benign and malignant etiologies. The real prevalence of all pleural pathologies is unclear in the literature. The most common primary malignancy of the pleura is a malignant mesothelioma, with an estimate of 10,000 new cases annually across Western Europe, Scandinavia, North America, Japan, and Australia [3]. Pleural metastases are the most common secondary malignancies often associated with malignant pleural effusion. They are mainly found in primary malignancies of the lung, breast, lymphoma, and ovarian cancer [4]. Primary pleural malignancies, other than mesothelioma, are less frequent and are often a diagnostic challenge for radiologists. According to the 2016 World Health Organization classification of tumors of the pleura, they can be divided into mesothelial tumors, lymphoproliferative disorders, and mesenchymal tumors [5].

This article focuses on primary pleural malignancies, other than mesothelioma, and juxtapleural malignancies. The aim of this pictorial review is to provide a structured overview of the imaging features, with clues for diagnosis and differential diagnosis (Table 1).

**Table 1:** Reviewed tumors and tumorlike conditions with an overview of the most important clinical characteristics and diagnostic signs

	Clinical characteristics	Radiological signs
**Primary pleural malignancies: lymphoproliferative disorders**		
Primary effusion lymphoma	Immune deficiency (HIV, Kaposi)	Isolated unilateral effusion
Pyothorax-associated lymphoma	Chronic pyothorax, EBV	Inhomogeneous solid pleural mass
**Primary pleural malignancies: mesenchymal tumors**		
Solitary fibrous tumors	Older patients, mainly asymptomatic, hypertrophic pulmonary osteoarthropathy, Doege–Potter syndrome (hypoglycemia)	Well-circumscribed heterogeneous mass, incomplete border sign, pedunculated, not locoregionally aggressive
Desmoid type fibromatosis	Mainly young patients, Gardner syndrome (familial adenomatous polyposis)	Well-defined homogeneous mass
Pleural sarcomas: synovial sarcoma	Aspecific	Well-defined heterogeneous mass
Pleural sarcomas: angiosarcoma	Predominantly male adults	Diffuse pleural thickening with unilateral effusion, rapid progression
Desmoplastic small round cell tumor	Young patients	Aspecific
**Juxtapleural malignancies**		
Chondrosarcoma and osteosarcoma	Elderly patients	Heterogeneous mass with bone destruction
Extraskeletal Ewing sarcoma	Children and adolescents, pain, swelling	Bone destruction and large soft-tissue component, sunburst sign
Leiomyosarcoma	Aspecific, hemoptysis	Aspecific
Neurogenic tumors: benign	Younger patients	Fascicular sign, target sign, vertebral scalloping
Neurogenic tumors: malignant	Neurofibromatosis type 1	Larger lesions (> 5 cm)
Liposarcomas	Asymptomatic (slow grow)	Fat containing lesion, inhomogeneous

## Imaging strategy

Conventional radiography (CR) is often the first-line imaging modality for patients with thoracic complaints. One of the signs of an extrapulmonary lesion on chest CR is an incomplete border sign, in which part of the border is invisible. Other signs suggestive of a pleural lesion are displacement of the pulmonary vasculature and respiratory-associated changes in location of the lesion [6]. However, chest CR is often aspecific. Pleural effusion is a common secondary finding that can also be diagnosed by ultrasound. The presence of fibrous septations and/or loculation of the pleural effusion is suggestive of a chronic or malignant effusion. Peripherally located lesions and locoregional chest wall and/or rib invasion may also be visualized using ultrasound [7, 8]. Computed tomography (CT) is used for further visualization and characterization of the pleural lesion, and to define its relationship with the surrounding structures [1]. When comparing to CR and CT, Magnetic resonance imaging (MRI) has a better ability to determine the relationship between the pleural lesion and the surrounding structures, in particular the chest wall and spinal canal. Diffusion-weighted imaging (DWI) on MRI may be helpful in differentiating benign from malignant pleural lesions and in correcting false-positive 18F-Fluorodeoxyglucose positron emission (18-FDG PET) CT findings [9, 10]. The 18F-FDG PET is indicated for the evaluation of distant metastases and other abnormalities that can lead to diagnosis. For complete diagnostic evaluation, ultrasound- or CT-guided biopsy of the lesion and/or pleural effusion may be necessary.

## Primary pleural malignancies

### Lymphoproliferative disorders

Pleural lymphomas are mainly seen as secondary when in association with other thoracic abnormalities or in cases of aggressive subtypes. They arise from the direct extension from a pulmonary or nodal disease, or through hematogenous or lymphatic dissemination. Patients present with aspecific complaints, including chest pain, dyspnea, or cough. Pleural effusion is one of the main manifestations on imaging. Pleural thickening or solid pleural lesions are less commonly seen with frequently missed pleural lesions due to small size or fluid superposition. Both unilateral and bilateral pleural involvements are possible [11, 12].

**Fig. 1:**
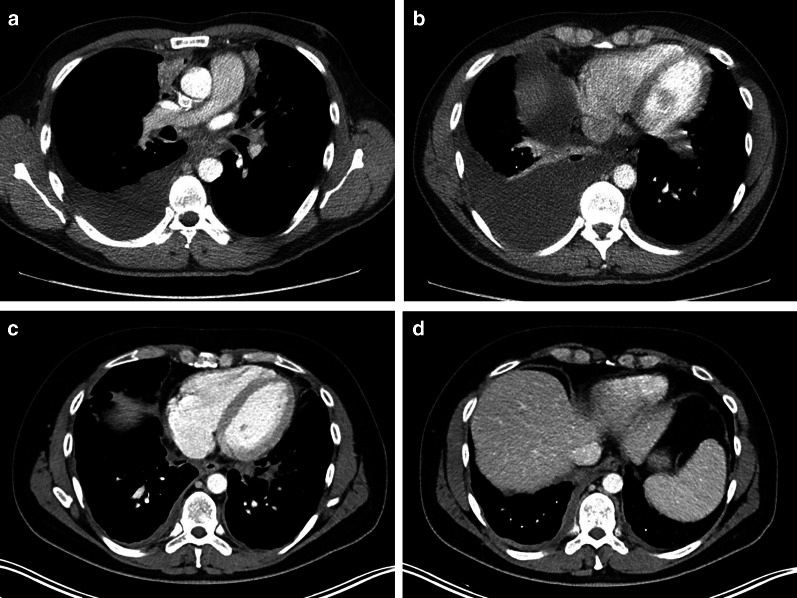
Diagnosis: primary effusion lymphoma. Technique: contrast-enhanced chest CT. Description: A 41-year-old man with a history of HIV, HHV8, and EBV presented with dyspnea and chest pain. The initial CT examination (**a**, **b**) shows a prominent unilateral pleural effusion posterior to the right lung. A combination of imaging and clinical findings was suggestive of primary effusion lymphoma, which was histopathologically confirmed. Follow-up CT after treatment (**c**, **d**) shows a small residual right-sided pleural effusion

Primary pleural lymphomas are rarely diagnosed and consist of two distinct subtypes [13]. The first subtype is the primary effusion lymphoma (PEL), which is a diffuse large B-cell lymphoma (DLBCL) caused by the human herpes virus type 8 or Kaposi sarcoma-associated herpes virus. A unilateral effusion with a homogeneous near-water attenuation is seen on imaging without evidence of solid disease. Therefore, an isolated pleural effusion in a patient with an AIDS-related immune deficiency should alert radiologists and clinicians to the possible diagnosis of PEL (Fig. 1).

The second subtype is the pyothorax-associated lymphoma (PAL). This Epstein-Barr virus (EBV)-positive DLBCL is exclusively seen in patients with chronic pyothorax, usually due to iatrogenic-induced pneumothorax from tuberculosis treatment [3]. The highest incidence of PAL is seen in Japan, as more than 90% of the population is infected with EBV, and lung collapse therapy is widely performed there [12]. Imaging characteristically shows an inhomogeneous solid pleural mass that may extend directly into the lung; locoregional rib destruction has been described in the literature. In contrast to PEL, pleural effusion is not always present. Both PEL and PAL are associated with a poor prognosis [7, 12].

### Mesenchymal tumors

The term mesenchymal tumors includes a broad spectrum of various pathologies originating from transformed cells of connective tissue origin. One of the characteristics of these primary malignancies is that most of them are focal.

#### Solitary fibrous tumors

Solitary fibrous tumors (SFTs) are the second most common primary pleural tumors following mesothelioma, although less than 800 cases have been reported in the literature. Patients from all ages have been described, but predominantly in the age range of 50 to 60 years [14]. Since up to half of patients are usually asymptomatic, SFT is often an incidental finding; however, cough, chest pain, and dyspnea may be presenting symptoms. In 20% of cases, the mass originates from the parietal pleura, predisposing to chest pain. SFTs may be benign or malignant, with approximately 10% to 20% of SFTs being malignant. Malignant SFTs are often associated with clinical symptoms; however, there is no correlation between symptoms and diagnosis of malignancy. SFT is associated with hypertrophic pulmonary osteoarthropathy in 10–30% of cases (Pierre-Marie-Bamberger syndrome). Rarely, patients present with hypoglycemia secondary to tumor-induced production of insulin growth factor (IGF) 2. The IGF-2 leads to activation of IGF-1 and an insulin-like response resulting in hypoglycemia. Doege–Potter syndrome is rare, with an estimated incidence of 3–4% [15].

**Fig. 2:**
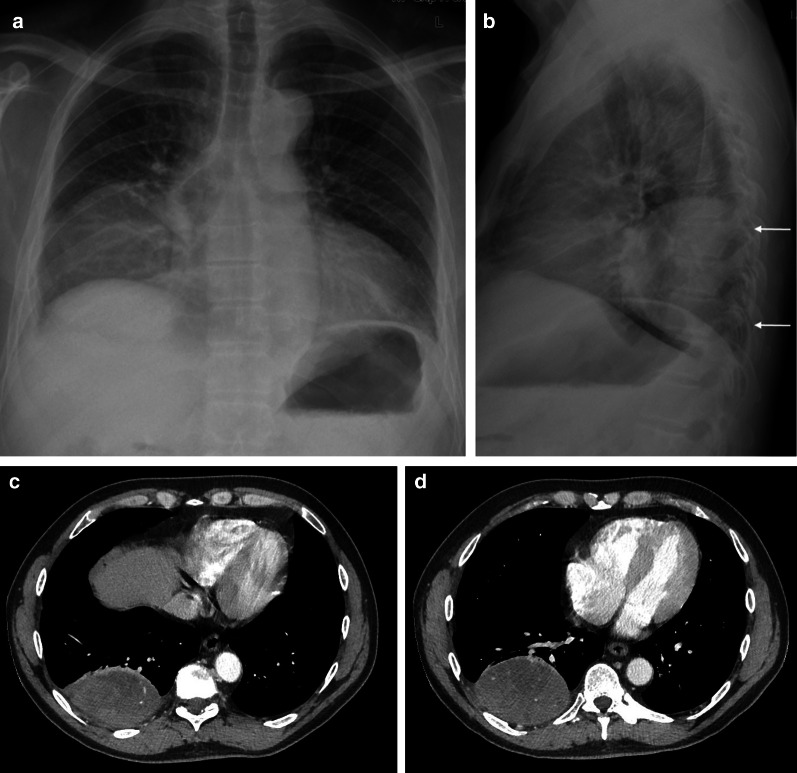
Diagnosis: solitary fibrous tumor. Technique: standard chest radiography and contrast-enhanced chest CT. Description: A 65-year-old man presented with acute right-sided posterior chest pain. There was no history of weight loss. Postero-anterior and lateral chest radiographs (**a**, **b**) show a well-circumscribed mass located posteriorly in the right lower lobe with an incomplete border sign (white arrows), suggestive of an extrapulmonary mass. Axial contrast-enhanced CT (**c**, **d**) shows a well-defined, heterogeneous mass with intralesional hypodense areas of necrosis and hyperdense calcifications. Imaging findings were suggestive of a solitary fibrous tumor. Histopathologic examination of the resected mass confirmed this diagnosis, showing signs of malignancy

**Fig. 3:**
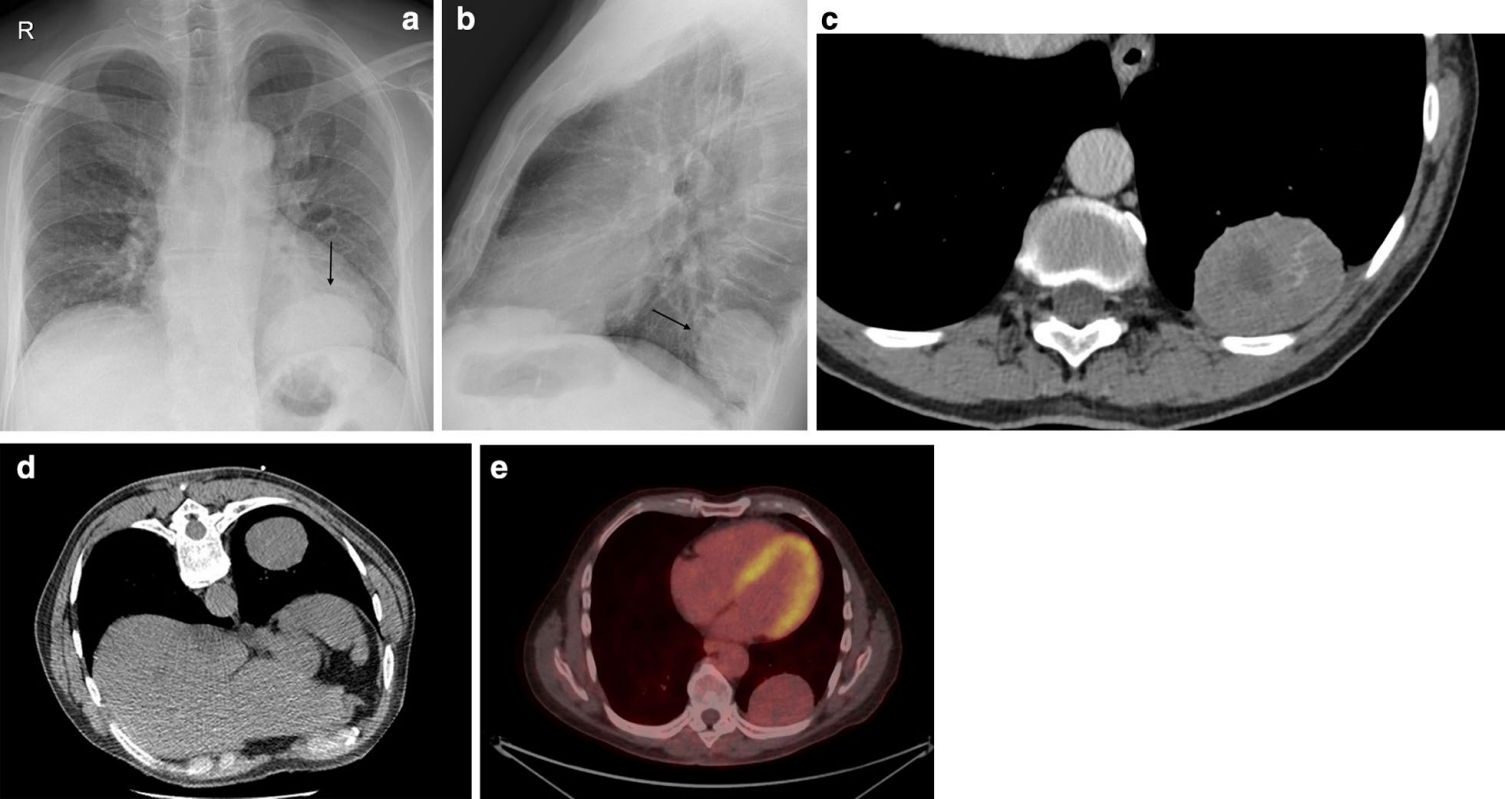
Diagnosis: solitary fibrous tumor. Technique: standard chest radiography, contrast-enhanced chest CT and 18FDG-PET CT. Description: A 64-year-old man was referred for a chest radiograph as part of the investigations for gastro-intestinal complaints. Postero-anterior and lateral radiographs (**a**, **b**) show as incidental finding, a well-delineated mass (arrow) located posteriorly in the left lower lobe. Axial contrast-enhanced CT (**c**) in the mediastinal window setting shows a well-delineated pleural based heterogeneous mass with intralesional hypodense foci and areas of more prominent contrast enhancement. There is a change in location of the tumoral mass on the axial CT in the prone position (**d**), which was performed as part of a CT-guided biopsy. This change in location is an indirect sign suggestive of a pedunculated tumor. The lesion does not show high uptake on 18-F-FDG PET imaging (**e**). Imaging characteristics were suggestive of a solitary fibrous tumor. This was confirmed by histopathologic examination of the resected mass

Standard chest radiography revealed a well-circumscribed pulmonary mass (Fig. 2) with variable size, located adjacent to the pleural surfaces or fissure. A pedunculated tumor may change location on sequential images. As the peduncle itself is not always visible, the change in location is an indirect sign, suggestive of a pedunculated tumor (Fig. 3).

CT is the imaging modality of choice and allows a detailed view of a well-circumscribed mass. SFTs are generally heterogeneous on CT (Fig. 2), with focal hypoattenuating areas due to necrosis, cystic degeneration, or myxoid components. Intralesional hemorrhage and calcifications (2 up to 26%) are seen as hyperdense foci. The focal mass effect leads to displacement of the surrounding structures rather than invading them [16].

**Fig. 4:**
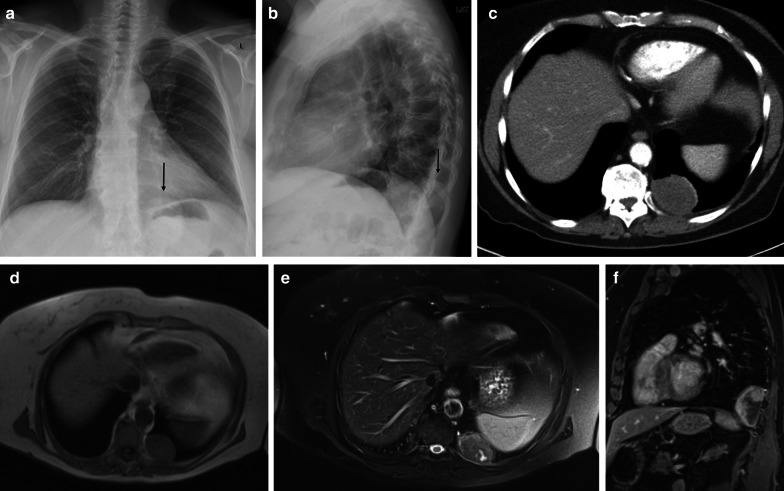
Diagnosis: solitary fibrous tumor. Technique: standard chest radiography, contrast-enhanced chest CT and MRI. Description: A 68-year-old woman presented with progressive complaints of dyspnea, cough, and limited weight loss. Postero-anterior and lateral radiographs (**a**, **b**) show a well-delineated mass (arrow) located posteriorly in the left lower lobe. Axial contrast-enhanced CT in the mediastinal window setting (**c**) shows a well-delineated homogeneous pleural-based mass. There is no invasion of the surrounding structures. On the axial T1-WI MR image (**d**), the lesion has a homogeneous isointense signal intensity to muscle. On the axial T2 blade FS MR image (**e**), the lesion has a low signal intensity with peripheral enhancement on the sagittal T2W FS MR image after i.v. contrast administration (**f**)

MRI can be used to evaluate the relationship between the chest wall and diaphragm. Typically, a low to intermediate signal intensity on T1-weighted images (WI), and low signal intensity (SI) on T2-WI are seen (Fig. 4). After intravenous gadolinium contrast administration, there is a heterogeneous enhancement of the mass.

The most diagnostic challenge in SFTs is the differentiation between benign and malignant tumors [17]. The most important imaging parameter is the size of the lesion; if it is larger than 10 cm, the SFT is malignant until proven otherwise [13, 16]. An increase in size over time, pleural effusion, chest wall invasion, and hypervascularity are other imaging features that may suggest malignancy [14, 17].

**Fig. 5:**
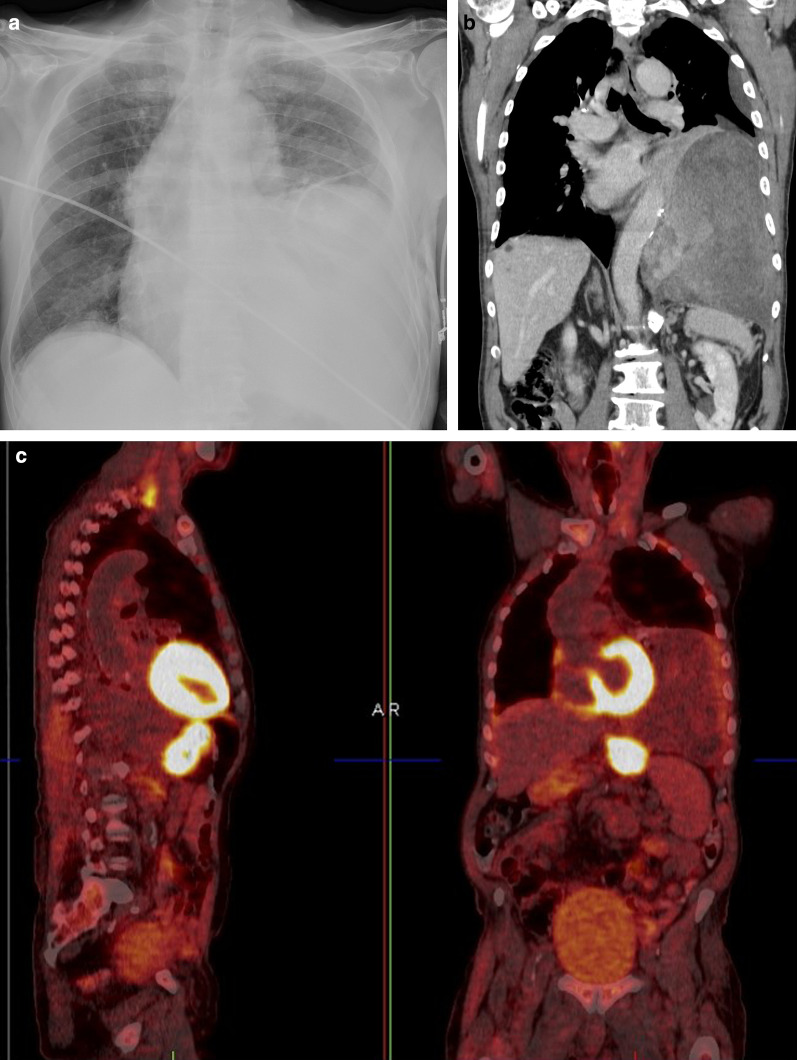
Diagnosis: solitary fibrous tumor. Technique: standard chest radiography, contrast-enhanced chest CT and 18FDG-PET CT. Description: An 84-year-old man presented to the emergency department with clinical symptoms of weight loss, altered behavior, and fever. His bloodwork showed signs of hypoglycemia. Standard antero-posterior chest radiograph (**a**) shows a large area of consolidation in the left hemithorax, with a silhouette sign on the left heart border and left diaphragm. Coronal reformatted contrast-enhanced CT-image (**b**) shows a well-defined mass in the left hemithorax. The mass has a heterogeneous aspect with intralesional hypodense areas due to necrosis, cystic degeneration or myxoid component. The mass does not show an aggressive growth pattern: there are no imaging signs that suggest invasion in the adjacent thoracic aorta. There is no increased 18F-FDG uptake in the tumoral mass on the 18-F-FDG PET study (**c**). The combination of imaging findings and clinical findings was suggestive of Doege–Potter syndrome, which was confirmed after histopathologic examination of the large resected pleural mass

Data on the use of 18-F-FDG PET imaging in SFTs are scarce, and FDG-PET seems to be of little value in the diagnosis of benign and malignant tumors and to differentiate between them. Malignant tumors tend to be more hypermetabolic than benign tumors, but there is an overlap in standardized uptake value (SUV) max values between these tumors [18]. 18F-FDG-PET may have a high negative predictive value in assessing the malignancy of SFTs (Fig. 5) [19].

#### Desmoid-type fibromatosis

**Fig. 6:**
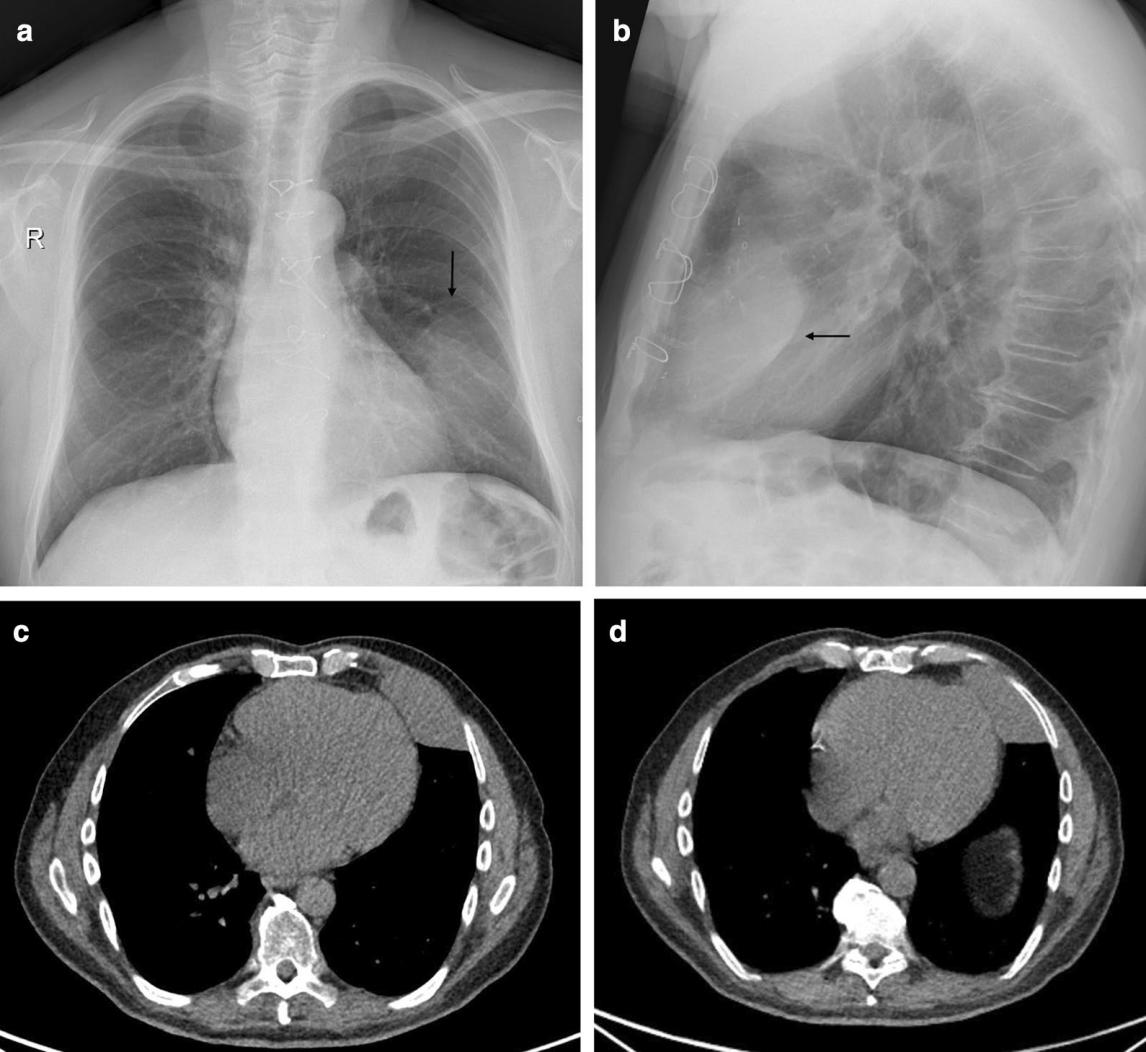
Diagnosis: desmoid tumor. Technique: standard chest radiography and chest CT. Description: A 76-year-old man (never smoker) presented with dyspnea and chronic cough. Postero-anterior and lateral chest radiographs (**a**, **b**) show a relatively ill-defined area of consolidation (arrow) in the left lung (lingular region). Axial non-contrast-enhanced CT (**c**, **d**) shows a relatively well-defined oval-shaped pleural-based mass, isodense to muscle. There are no signs of associated chest wall involvement. Diagnosis of a desmoid tumor was made after surgical resection

Desmoid-type fibromatosis, also known as aggressive fibromatosis, is a benign disease characterized by fibroblast proliferation with an infiltrative character. Although these tumors are not intrinsically malignant, they are often classified as such because of their infiltrative character and high risk of recurrence after surgical resection. Desmoid tumors are predominantly seen in young patients with a female-to-male ratio of 2:1 [20]. An underlying genetic mutation of the adenomatous polyposis of the colon (APC) gene is found in patients with Gardner’s syndrome or familial adenomatous polyposis (FAP). The intrathoracic location is the second most frequent location in addition to the primary intra-abdominal location. In 15% of cases, there is multiplicity of the disease [21]. Most patients are asymptomatic, often with a large sized lesion as a coincidental finding on chest radiographs. On CT, which is the preferred imaging modality, lesions tend to present as well-defined, homogeneous solitary pleural masses that are isodense to muscle (Fig. 6). There are no intralesional hemorrhagic or necrotic foci. MRI shows a signal intensity iso-intense to muscle on T1-WI, whereas on T2 the signal varies depending on the characteristics of the lesion. A high T2-SI is seen in lesions with a high cellularity or myxoid component, whereas low SI is found if lesions predominantly contain collagen. Contrast enhancement is variable in both CT and MRI imaging [22–24].

#### Pleural sarcomas

Pleural sarcomas are most frequently encountered in the context of metastases and rarely as primary pleural sarcomas.

**Fig. 7:**
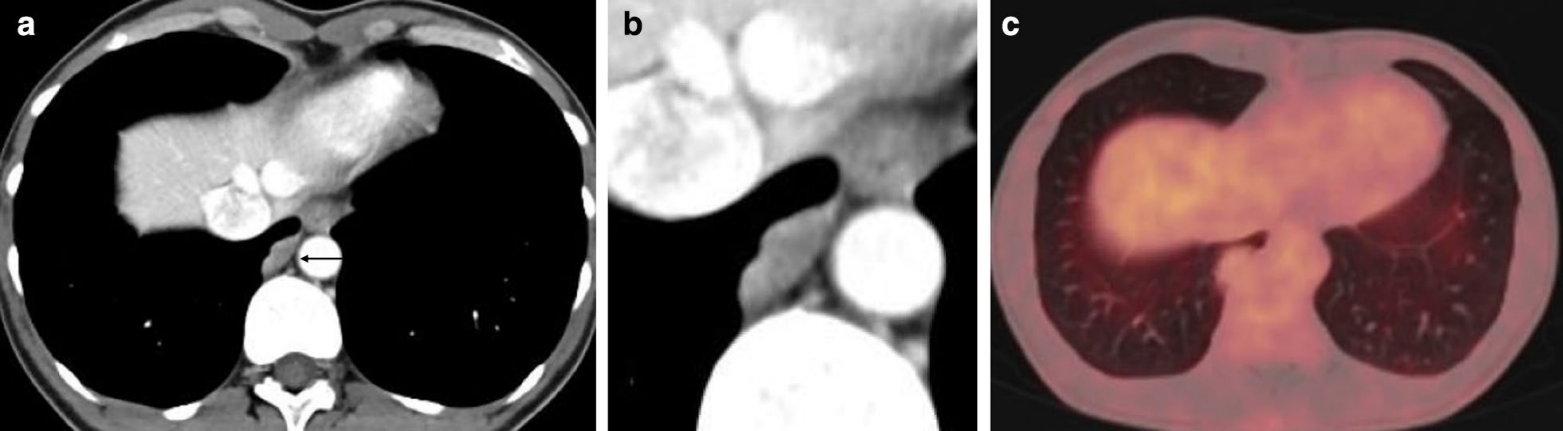
Diagnosis: synovial sarcoma. Technique: contrast-enhanced chest CT and 18FDG-PET CT. Description: A 43-year old woman with a history of a synovial sarcoma in the sartorius muscle was referred for a chest CT to rule out metastatic disease. Axial contrast-enhanced CT (**a**) shows a lobulated, well-defined pleural lesion (arrow) in the base of the right lung, which is slightly heterogeneous (see detail image **b**). There is no increased FDG uptake on the 18-F-FDG PET examination (**c**). Histopathologic examination of the resected specimen confirmed the diagnosis of synovial sarcoma

Synovial sarcomas are observed in patients with a wide age range and are mainly found as soft tissue sarcomas of the extremities. Primary pleural presentations are rare. On imaging studies, they manifest as a well-defined heterogeneous mass with intralesional necrosis or hemorrhage (Fig. 7), and lesions may vary in size. After contrast administration, a heterogeneous enhancement is observed. Associated pleural effusion and cortical bone destruction may also occur. Findings on 18-F-FDG PET are aspecific since FDG uptake may be variable [25]. The morphological features of primary and metastatic synovial sarcomas are similar and not differentiable on imaging.

**Fig. 8:**
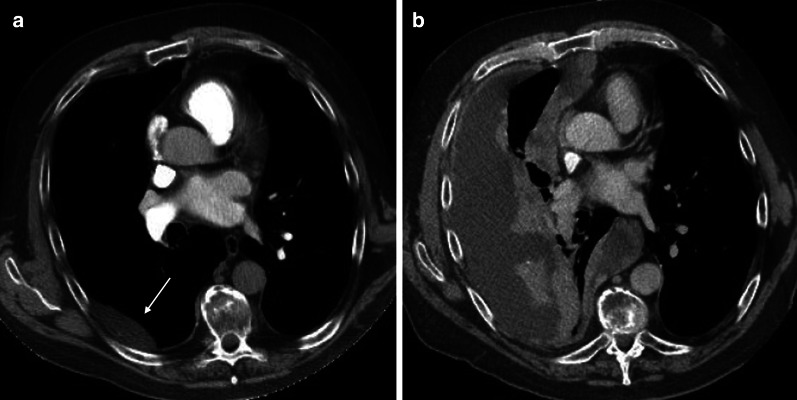
Diagnosis: angiosarcoma. Technique: contrast-enhanced chest CT. Description: A 64-year-old man with no history presented with progressive shortness of breath. The initial contrast-enhanced CT scan (**a**) to rule out pulmonary embolism shows a well-delineated pleural-based oval lesion (arrow) in the base of the right lung. Findings are relatively aspecific. Follow-up CT examination 4 weeks later (**b**) shows prominent disease progression, with a large amount of fluid. Despite the relatively short time interval, there is a marked increase in pleural lesions, with widespread focal contrast enhancing pleural lesions in the right hemithorax. The rapid and aggressive progression points to the possible diagnosis of angiosarcoma, which was histologically confirmed

Angiosarcomas are predominantly observed in adult males. They range from low-grade epithelioid hemangioendotheliomas (EHEs) to high-grade angiosarcomas and are typically very aggressive, especially in the high-grade form. EHE is a rare vascular tumor that may present at different sites in the body. Only 200 cases of pulmonary EHE have been reported worldwide, and only 27 have been classified as pleural in origin [26]. Patients are typically male and symptomatic at presentation. Chest pain, dyspnea, and cough are aspecific symptoms, but if persistent, they should be included in the differential diagnosis. Chest imaging shows pleural thickening with a unilateral pleural effusion [27]. This unilateral effusion should raise suspicion if it does not respond to therapy. An angiosarcoma may mimic a mesothelioma on imaging with diffuse pleural thickening (up to 40%) and pleural effusion (up to 70%) (Fig. 8) [28, 29]. Owing to its aggressive course, the prognosis is very poor.

#### Desmoplastic small round cell tumor

The etiology of desmoplastic small round cell tumor (DSRCT), a rare tumor, is believed to be a genetic alteration of two different tumors (Wilms tumor and Ewing’s tumor). This may explain the young age of the affected population. The clinical presentation is aspecific. DSRCT can present as a single lesion or as diffuse pleural involvement with pleural effusion. This diversity of presentation patterns can be attributed to the dual genetic alterations. Locoregional bone invasion has been described as an associated finding. Imaging findings are aspecific and impose a diagnostic challenge. In general, surgical biopsy is needed for the definite diagnosis of DSRCT [30, 31].

## Juxtapleural malignancies

Juxtapleural tumors are closely related to the pleura and chest wall and may originate from osseous structures, cartilage, soft tissue, muscles, nerves, or fat. Although imaging findings may overlap or be aspecific, some tumors may present with a particular imaging feature or specific clinical setting.

### Chondrosarcoma and osteosarcoma

**Fig. 9:**
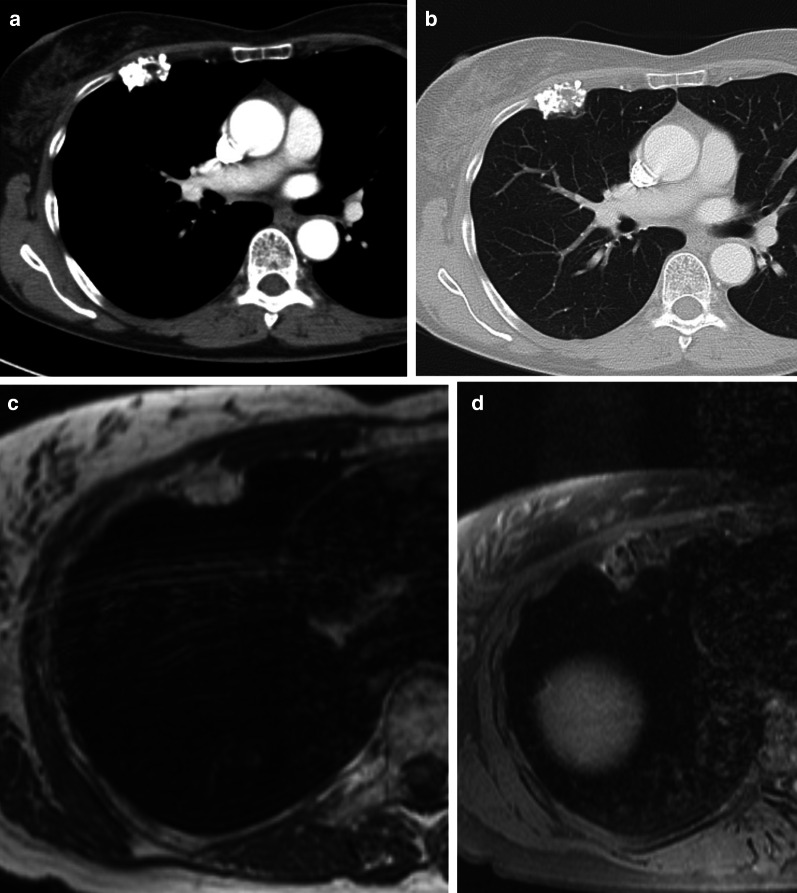
Diagnosis: chondrosarcoma. Technique: contrast-enhanced chest CT and MRI. Description: A 64-year-old woman with a history of smoking, presented due to shortness of breath. The axial contrast-enhanced CT scan (**a**, **b**) reveals an expansile lesion with extensive calcifications located at the costochondral junction of the right 3rd rib (note a typical arc-and-ring pattern of calcification). On the axial T1-weighted MR image (**c**) the lesions have a high signal intensity. The axial T2-weighted MR image (**d**) shows a peripheral hyperintense mass with internal low-intensity foci, reflecting the cartilaginous matrix with chondroid calcifications

**Fig. 10:**
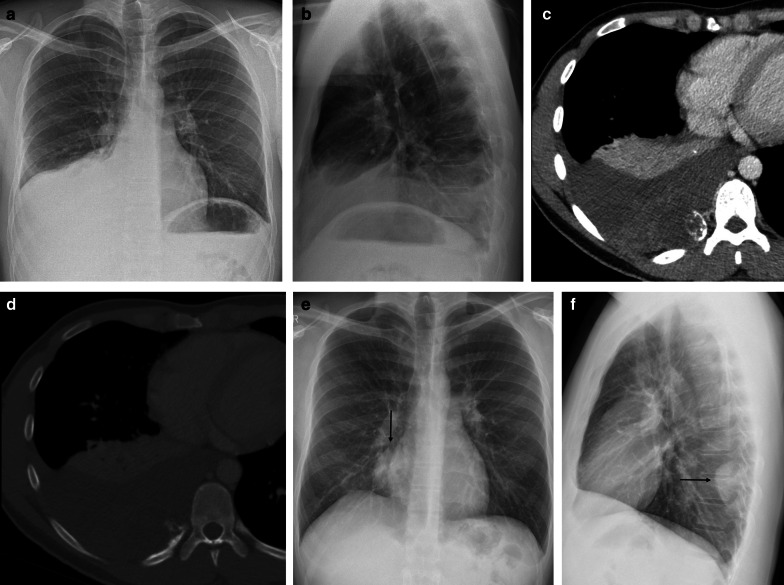
Diagnosis: chondrosarcoma. Technique: standard chest radiography and contrast-enhanced chest CT. Description: A 25-year-old male presented with progressive worsening, inspiration-bound thoracic pain. Postero-anterior and lateral chest radiographs (**a**, **b**), which were performed to rule out a spontaneous pneumothorax, show a unilateral right-sided pleural effusion. Further diagnostic work-up with CT was performed. Axial contrast-enhanced CT (**c**) shows a massive pleural effusion masking a heterogeneous tumoral mass arising from the posterior side of the 10th rib with arc-and-ring calcifications. The locoregional bone destruction is best seen in the bone window (**d**). The postero-anterior and lateral chest radiographs after drainage of the pleural fluid (**e**, **f**) reveal the nodular mass (arrow) projecting into the right paravertebral on the PA image and posterior in the right lower lobe on the lateral chest radiograph. These imaging characteristics were suggestive of a chondrosarcoma, which was histopathologically confirmed after surgical resection

Chondrosarcoma is the most common primary tumor of the chest wall and is most commonly seen in elderly patients. Chondrosarcomas are mainly located anteriorly in the chest wall and in the upper five ribs (Fig. 9). Chondrosarcomas produce chondroid matrices, and CT shows the typical imaging features, such as arc-and-ring sign or flocculence appearance (Fig. 10).

**Fig. 11:**
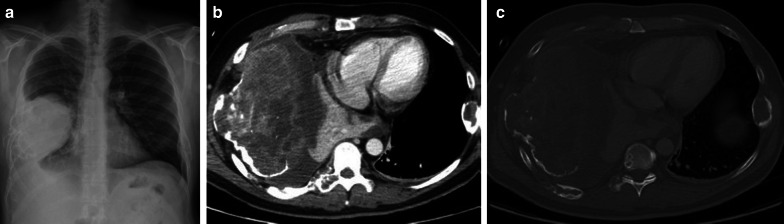
Diagnosis: osteosarcoma. Technique: standard chest radiography and contrast-enhanced chest CT. Description: A 50-year-old man presented with complaints of right-sided chest pain. The postero-anterior chest radiograph (**a**) shows a large mass located at the lateral side of the right hemithorax with an associated pleural effusion. Note also a lytic expansion of the 8th left rib. The axial contrast-enhanced CT in the mediastinal window setting (**b**) confirms the heterogeneous mass with intralesional necrosis and calcifications originating from the 7th right rib, with focal expansive destruction, best seen in the bone window (**c**). There is a known pleural effusion with adjacent compression atelectasis of the right lower lobe. Histopathologic examination of the large mass confirmed the diagnosis of an osteosarcoma. The expansive tumorlike lytic lesion of the 8th left rib corresponds with fibrous dysplasia

Osteosarcomas are primarily found in long bones; chest wall location is rare and is mainly observed in an elderly population. Osteosarcomas contain new neoplastic bone and/or disorganized ossification. Imaging characteristically reveals a heterogeneous mass with associated bone destruction (Fig. 11). Heterogeneity in imaging studies is due to intralesional necrosis, hemorrhage, and ossification. The pathognomonic sunburst sign is less evident in flat bones, thus hampering diagnosis [32].

### Extraskeletal Ewing sarcoma

**Fig. 12:**
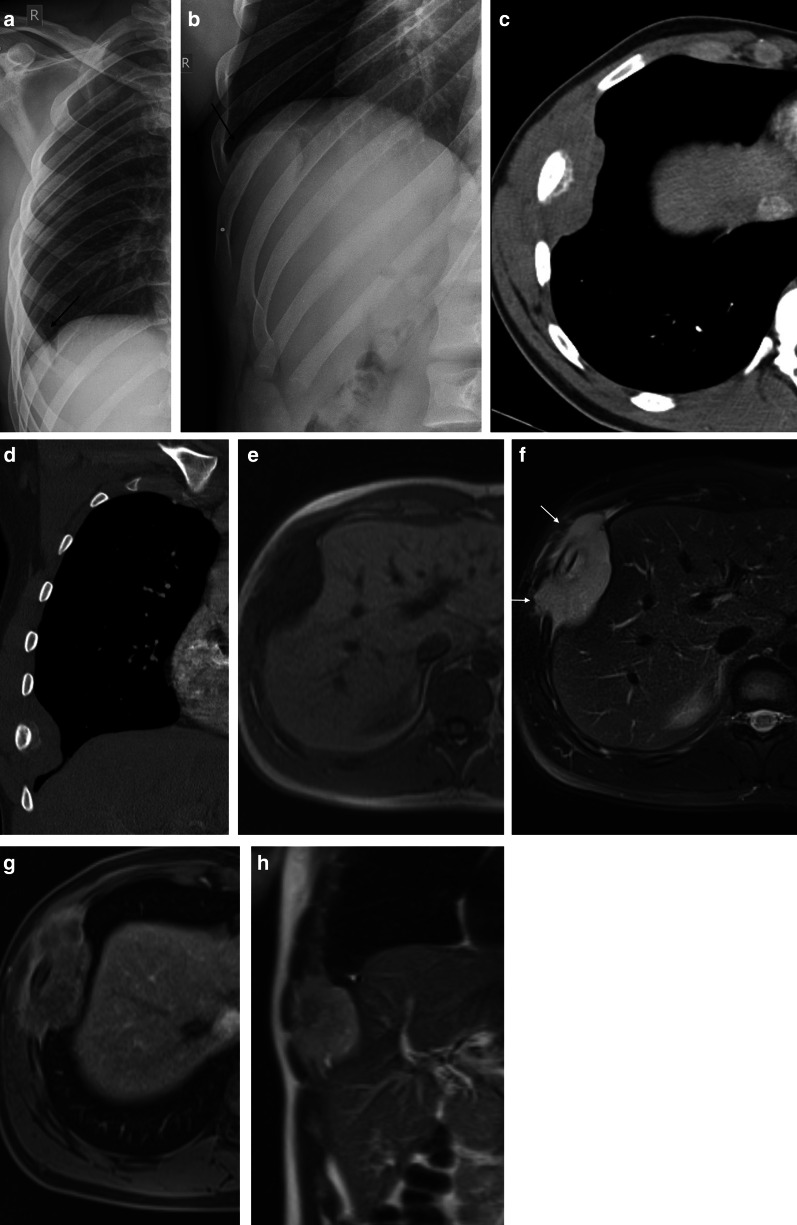
Diagnosis: Ewing sarcoma. Technique: standard chest radiography and contrast-enhanced chest CT. Description: A 23-year-old man was referred for a chest radiograph due to pain at the right hemithorax. The postero-anterior chest radiographs (**a**, **b**) show an opacity (arrow) projecting at the level of the 7th right rib. An external radio-opaque marker was placed on the painful area. The additional contrast-enhanced CT further characterizes the mass encasing the 7th right rib with a locoregional aggressive periosteal sunburst type reaction and a surrounding soft tissue component (**c**, **d**). On the axial T1-weighted MR image (**e**) the lesion has an isointense signal to muscle and a high signal intensity on the axial FS T2-WI (**f**) with invasion of the muscles of the chest wall (arrows). After intravenous contrast administration, the lesions show a heterogeneous enhancement (**g**), predominantly at the periphery of the lesion. The coronal T2-weighted MR image (**h**) shows the extrinsic impression of the lesion on the liver

**Fig. 13:**
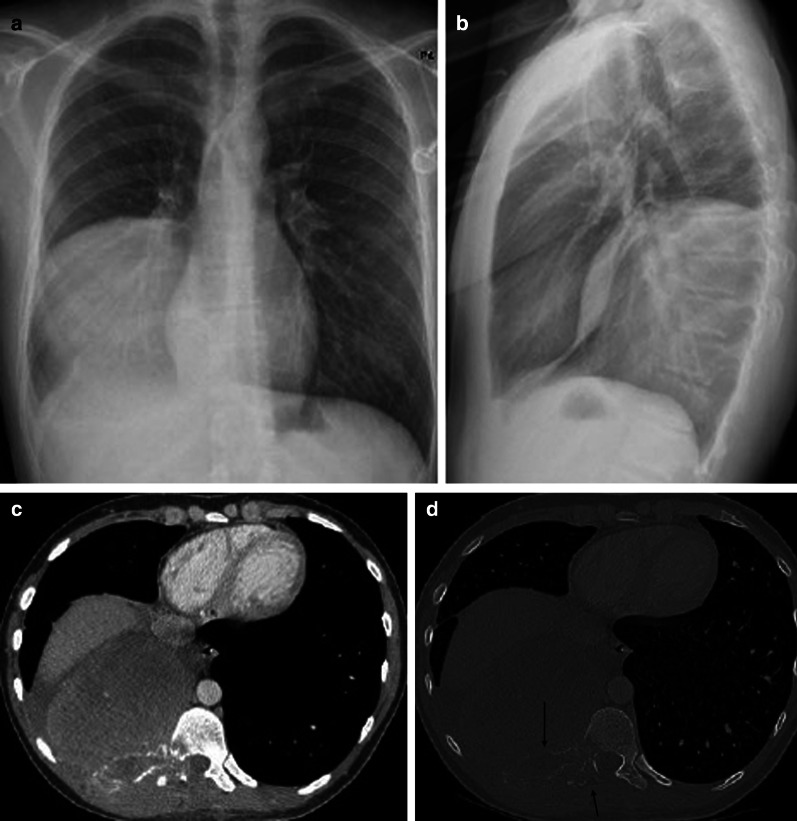
Diagnosis: extraskeletal Ewing sarcoma. Technique: standard chest radiography and contrast-enhanced chest CT. Description: A 17-year-old man presented with localized right-sided chest pain. Postero-anterior and lateral chest radiographs (**a**, **b**) show a well-delineated mass located posterior in the right lung. Axial contrast-enhanced CT (**c**) in the mediastinal window setting shows a heterogeneous mass with intralesional necrosis and focal bony destruction of the corpus of the vertebra, pedicle, and the posterior side of the rib (arrows), best seen in the bone window (**d**). The young age of the patient, locoregional bone destruction, and extensive soft tissue component is suggestive of an extraskeletal Ewing sarcoma

The second most common primary malignant bone tumor in children and adolescents is the Ewing sarcoma, with a slight male predilection (male-to-female ratio of 1.5:1). The primary complaint of patients is pain, which depends on the location, the feeling of a mass, or swelling. Ewing sarcoma can occur in extraskeletal sites in the chest, such as pleural and paravertebral sites. Most lesions have an aggressive appearance with locoregional bone destruction and a large soft-tissue component (Figs. 12, 13) [33].

### Leiomyosarcoma

**Fig. 14:**
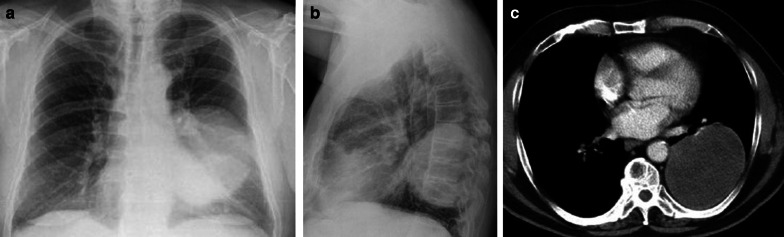
Diagnosis: leiomyosarcoma. Technique: standard chest radiography and contrast-enhanced chest CT. Description: Postero-anterior and lateral chest radiographs (**a**, **b**) in a 64-year-old man show a well-delineated mass located posteriorly in the left lower lobe. Axial contrast-enhanced CT (**c**) reveals a smooth lobular mass with intralesional necrosis. There is no locoregional invasion of the adjacent structures. Imaging findings are relatively aspecific. On histopathologic examination, findings were consistent with a leiomyosarcoma

A leiomyosarcoma arises from the smooth muscles and can be located adjacent to the bronchus, pulmonary artery, or peripheral pulmonary parenchyma. Depending on the site, it may evoke dyspnea, chest pain, or hemoptysis. Imaging shows a well-defined smooth or lobular mass with a homogeneous or necrotic aspect (Fig. 14) [32].

### Neurogenic tumors

**Fig. 15:**
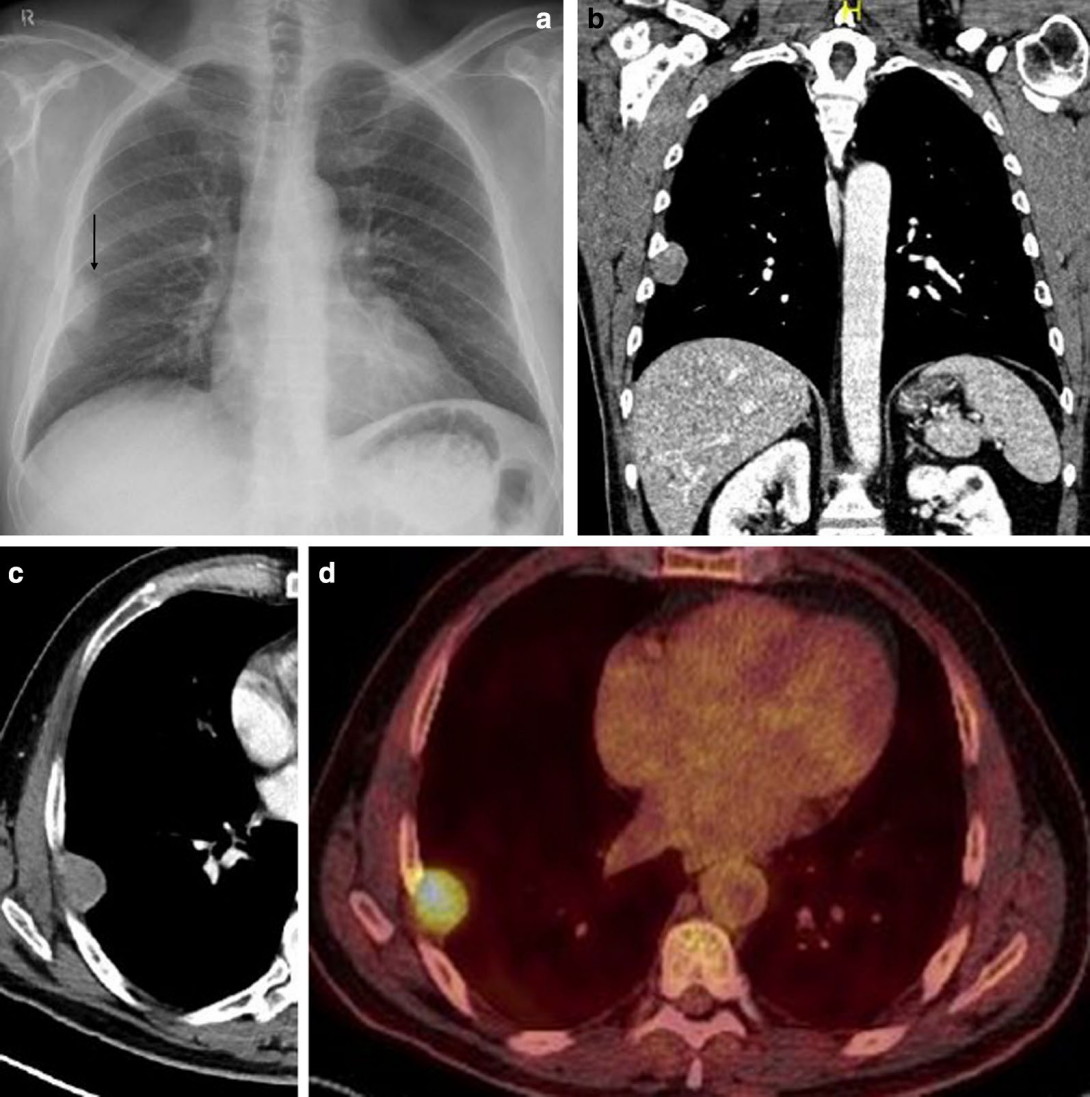
Diagnosis: schwannoma. Technique: standard chest radiography, contrast-enhanced chest CT and 18FDG-PET CT. Description: A 55-year-old man with no significant medical history was referred for a chest radiograph because of a persistent cough. As an incidental finding, a sharp delineated mass (arrow) laterally in the right hemithorax was found on the postero-anterior chest radiograph (**a**). Coronal and axial CT images (**b**, **c**) in the mediastinal window setting reveal a small homogeneous lesion located intercostally between the 5th and 6th right ribs. The lesion shows high uptake on 18-F-FDG PET (**d**). Although the high uptake on PET may mislead to a malignant cause, histopathologic examination of the resected specimen confirmed the suggested diagnosis of a schwannoma

**Fig. 16:**
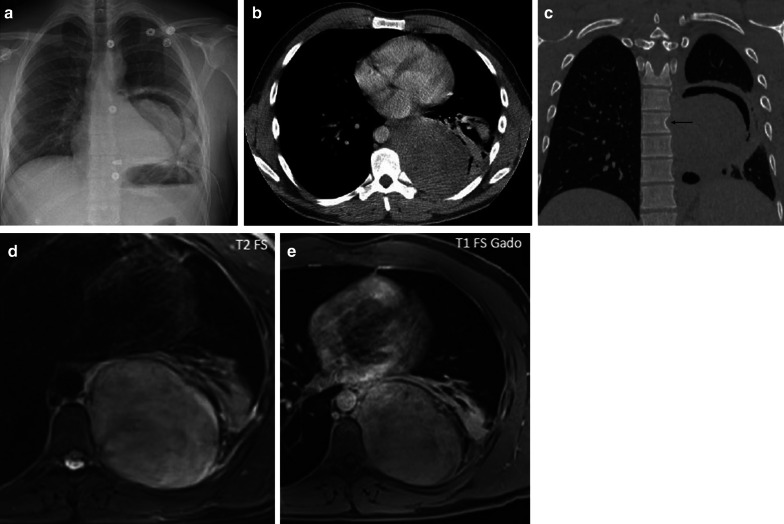
Diagnosis: benign schwannoma with low mitotic activity. Technique: standard chest radiography, contrast-enhanced chest CT and MRI. Description: A 55-year-old man with respiratory distress was transferred to our hospital after drainage of a hemothorax. The antero-posterior chest radiograph (**a**) shows a large mass in the left lung with a perilesional rim of air (due to pneumothorax). On contrast-enhanced CT (**b**) the homogeneous hypointense mass is located paravertebral in the left lower lobe with vertebral scalloping (arrow on **c**). There are no intralesional calcifications or necrosis. Note also the adjacent consolidation due to atelectasis. On the axial T2 FS WI MR image (**d**), the lesion has a high signal intensity and is relatively enhancing after contrast administration (**e**). Based on imaging findings and the size of the lesion, a malignant nerve sheath tumor was suggested. Histopathologic examination of the resected mass, however, did not reveal signs of malignancy but showed an 11-cm large benign schwannoma with low mitotic activity. Genetic counseling for NF-1 was negative

Neurogenic tumors with juxtapleural locations may arise from spinal nerve roots or intercostal nerves (Fig. 15). Benign neurogenic tumors, such as schwannomas and neurofibromas, are commonly diagnosed in younger patients (20–50 years old). Both show iso- to hypointense signal intensity on T1-WI. T2-weighted imaging is more distinctive with a fascicular sign (hypointense foci within the T2 hyperintense area) suggestive of a schwannoma, and a pathognomonic target sign (central hypointense and peripheral hyperintense SI) often seen in cases of neurofibromas. Benign schwannomas may show high uptake on PET, mimicking a malignancy. Malignant peripheral nerve sheath tumors (MPNSTs) are not as common and comprise 5–10% of all soft tissue sarcomas. More than 50% of the cases are associated with neurofibromatosis type 1. Lesions tend to be large (> 5 cm) with ill-defined margins and rapid interval growth on imaging studies. Size is the major discriminant between benign and malignant lesions; the larger the lesion, the more likely it is to be malignant. On MRI, they manifest with an iso- to hyperintense SI on T1-WI and a heterogeneously hyperintense SI on T2-WI due to intralesional necrosis and hemorrhage (Fig. 16) [25, 32, 34]. On 18F-FDG-PET, benign schwannomas may show an elevated FDG uptake [35].

### Liposarcomas

Liposarcomas are the malignant counterparts of the frequently found lipomas. Depending on the size, patients may present with thoracic complaints and discomfort. As these lesions grow slowly, patients mostly remain asymptomatic for a long period. [36] The diagnosis is often made on CT with typically a fat-containing lesion with negative Hounsfield units, often inhomogeneous and poorly distinctive from the surrounding structures. Intralesional calcifications may also be observed. On MR imaging, the fatty components have a signal intensity similar to fat on all pulse sequences with intralesional thick septa (> 2 mm) and contrast enhancement [32].

## Mimickers

**Fig. 17:**
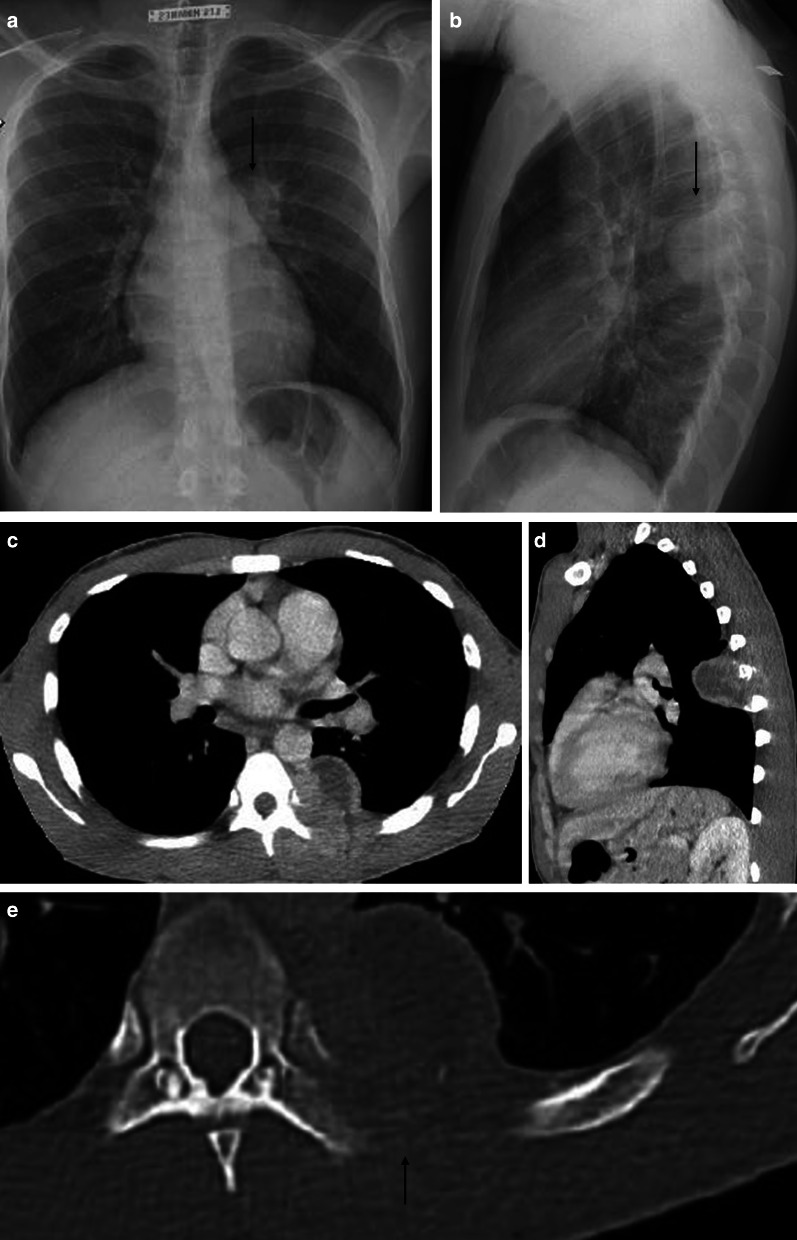
Diagnosis: tuberculous cold abscess. Technique: standard chest radiography and contrast-enhanced chest CT. Description: A 26-year-old man with marijuana abuse presented with acute back pain of two-week duration. There was no weight loss, no night sweats or fever. Postero-anterior and lateral chest radiographs (**a**, **b**) show a well-delineated mass-like lesion posterior in the left lung. Contrast-enhanced CT in the mediastinal window setting (**c**, **d**) shows a heterogeneous mass with intralesional necrosis and focal bone destruction (arrow) of the adjacent rib, best seen in the bone window (**e**). Transthoracic CT-guided biopsy was performed. Histopathologic examination of the specimen confirmed the diagnosis of a tuberculous cold abscess

**Fig. 18:**
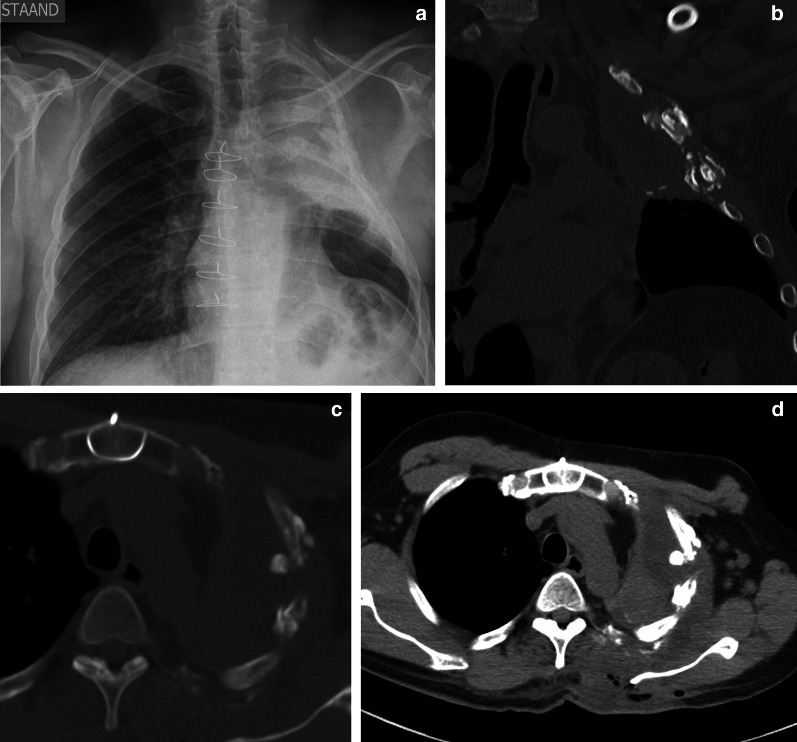
Diagnosis: radio-osteonecrosis and/or osteomyelitis. Technique: standard chest radiography and contrast-enhanced chest CT. Description: A 43-year-old man presented with left-sided chest pain. His medical history includes a lobectomy of the left upper lobe and radiotherapy. The postero-anterior chest radiograph (**a**) shows deformity and volume loss of the left hemithorax with a dense aspect of the lobectomy space. The coronal and axial CT thorax in bone window (**b**, **c**) reveals extensive periosteal reaction of the left ribs with inhomogeneous osteosclerosis and necrotic bone (with a typical denser aspect). The mediastinal window setting (**d**) is better to evaluate the adjacent soft tissue component and the fluid in the lobectomy space

**Fig. 19:**
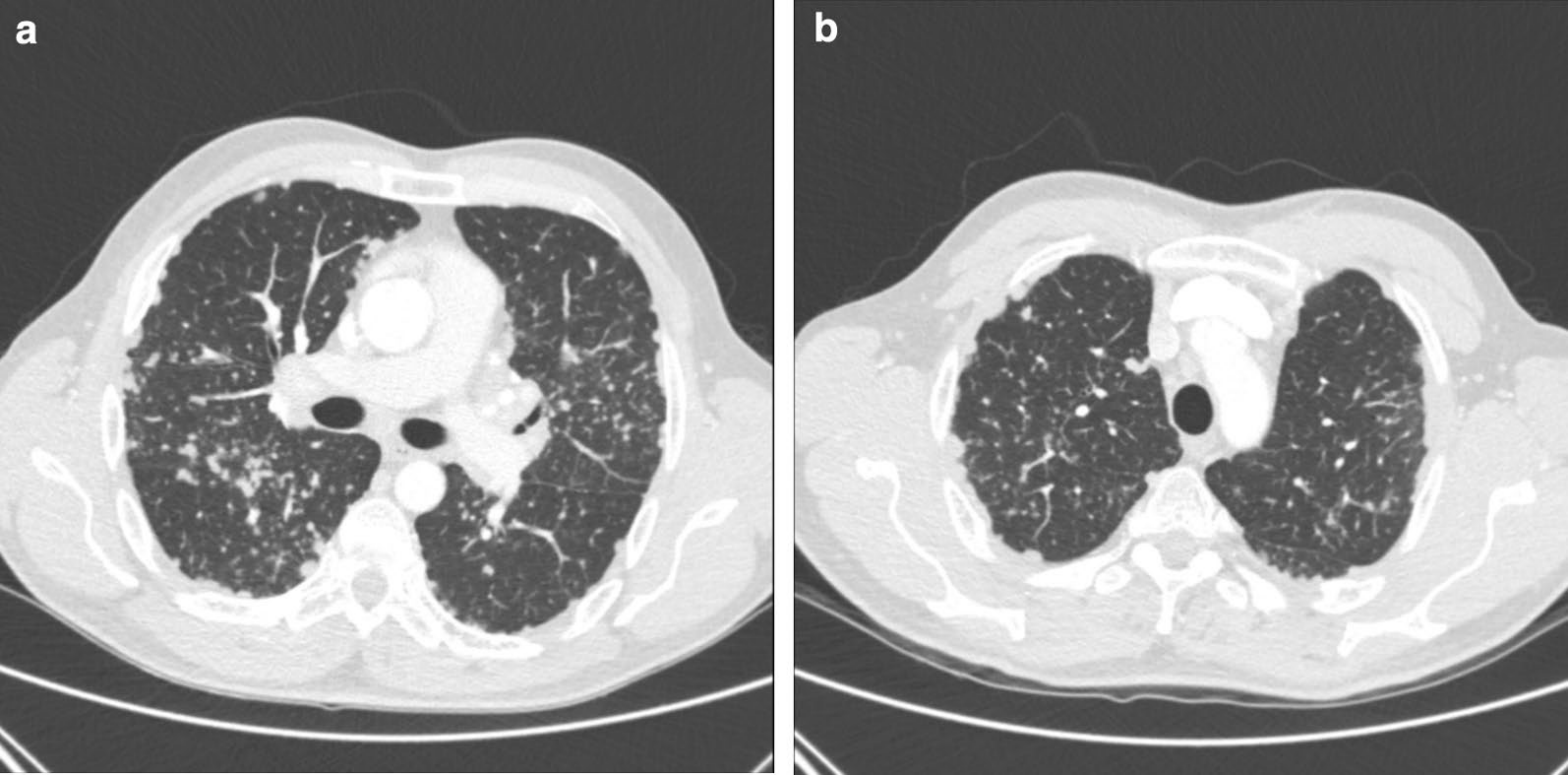
Diagnosis: pleural sarcoidosis. Technique: axial contrast-enhanced chest CT. Description: A 50-year-old man was referred for a chest CT as part of an investigation for shortness of breath. The axial contrast-enhanced CT in lung window settings (**a**, **b**) shows extensive bilateral nodular pleural thickening with diffuse micronodules. Video-assisted thoracoscopy was performed and histopathologic examination showed findings consistent with pleural sarcoidosis

**Fig. 20:**
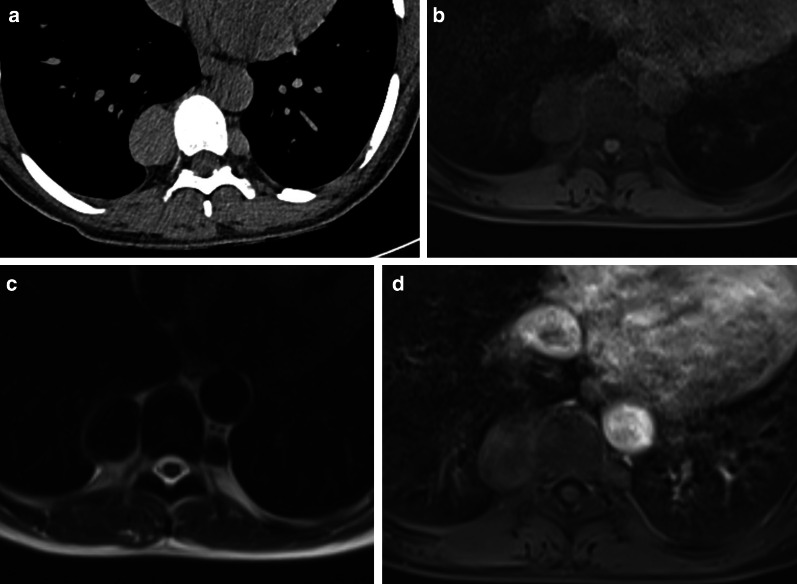
Diagnosis: extramedullary hematopoiesis. Technique: chest CT and MRI. Description: A 42-year-old woman with sickle cell anemia was referred for a routine control examination. The axial CT in the mediastinal window setting (**a**) shows as incidental findings, two well-delineated paravertebral masses with a homogeneous hypodense aspect. There is no locoregional bone invasion. On both the axial T1 vibe MRI sequence with FS (**b**) and the axial T2 haste MRI sequence (**c**), the lesions have the same signal intensity as bone marrow. There is no contrast enhancement of the lesions on the axial T1 vibe sequence with FS (**d**)

A number of infectious diseases may present as tumors or tumorlike pleural or juxtapleural diseases, mimicking a malignancy, for example, a chest wall cold abscess caused by tuberculosis. This is a rare entity, often simulating a pleural tumor when seen in the chest wall region. Patients may present with thoracic pain, but the pathognomonic clinical symptoms of tuberculosis, such as fever, weight loss, or night sweats, are not always present. Imaging shows a well-defined lobular mass against the pleura (Fig. 17), usually a solitary lesion. The mass may involve the ribs, vertebrae, costochondral junction, or sternum. In most cases, a biopsy is required to confirm the diagnosis [37]. Osteomyelitis (Fig. 18) is another infectious pathology that may mimic a pleural/juxtapleural malignancy. It is mainly seen in young children (< 5 years) and adults (bimodal age distribution). The clinical symptoms are aspecific and include pain, erythema, and edema of the affected body part. Standard radiography is a baseline examination for follow-up and differential diagnosis. MRI is the preferred modality for early detection, whereas CT may be useful in the evaluation of chronic osteomyelitis, particularly in areas with complex anatomy [38]. Tumorlike conditions of the pleura can be focal, but in general, they tend to be multifocal. Conditions such as pleural plaque, thoracic splenosis, thoracic endometriosis, Erdheim-Chester disease, pleural sarcoidosis (Fig. 19), and extramedullary hematopoiesis (Fig. 20) may mimic primary pleural malignancies. Extramedullary hematopoiesis must be in the differential diagnosis when sharply delineated paraspinal masses with a homogeneous signal intensity following bone marrow are encountered on MRI in a patient with a myeloproliferative disorder or hemoglobinopathy [39]. Interpretation of imaging findings in the clinical context is of utmost importance for a correct diagnosis.

## Conclusion

Pleural lesions are a diagnostic challenge for radiologists. Patient characteristics, such as age, immune status, hypoglycemia, and previous medical history, can guide the radiologist to the correct diagnosis. Although lesions can be initially detected on chest radiographs, CT and MRI are the imaging modalities of choice for further characterization. Imaging findings can be relatively specific in a number of cases, such as in SFT (presenting as a large well-defined and local non-aggressive mass), osteosarcoma (an aggressive lesion closely related to bone), or chondrosarcoma (location and chondroid matrix). In general, imaging findings are rather aspecific. Evolution is an important clue for the diagnosis. Additional extrathoracic imaging findings such as lymphadenopathies, organomegaly, and polyposis of the colon are important for accurate diagnosis. Discussion on a multidisciplinary thoracic oncology tumor board is vital for different steps in the diagnostic process, from detection on imaging studies to guiding the diagnostic approach (e.g., tissue prelevation), histopathologic diagnosis, and defining the treatment strategy.

## Abbreviations

18F-FDG PET: 18F-Fluorodeoxyglucose positron emission tomography
APC: Adenomatous polyposis of the colon
CR: Conventional radiography
CT: Computed tomography
DLBCL: Diffuse large B-cell lymphoma
DSRCT: Desmoplastic small round cell tumor
DWI: Diffusion-weighted imaging
EBV: Epstein-Barr virus
EHE: Epithelioid hemangioendothelioma
FAP: Familial adenomatous polyposis
IGF: Insulin growth factor
MRI: Magnetic resonance imaging
PAL: Pyothorax-associated lymphoma
PEL: Primary effusion lymphoma
SFT: Solitary fibrous tumors
SI: Signal intensity
SUV: Standardized uptake value
WI: Weighted images
